# Efficacy of Vonoprazan vs. Intravenous Proton Pump Inhibitor in Prevention of Re-Bleeding of High-Risk Peptic Ulcers: A Randomized Controlled Pilot Study

**DOI:** 10.3390/jcm13123606

**Published:** 2024-06-20

**Authors:** Tanawat Pattarapuntakul, Thanawin Wong, Panu Wetwittayakhlang, Nisa Netinatsunton, Suriya Keeratichananont, Apichat Kaewdech, Sawangpong Jandee, Naichaya Chamroonkul, Pimsiri Sripongpun, Peter L. Lakatos

**Affiliations:** 1 Gastroenterology and Hepatology Unit, Division of Internal Medicine, Faculty of Medicine, Prince of Songkla University, Hat Yai, Songkhla 90110, Thailand; tanawat_kuey@hotmail.com (T.P.); wongthanawin68@gmail.com (T.W.); apichat.ka@psu.ac.th (A.K.); tekikung@gmail.com (S.J.); naichaya@gmail.com (N.C.); spimsiri@medicine.psu.ac.th (P.S.); 2 Division of Gastroenterology and Hepatology, McGill University Health Centre, Montreal, QC H3G 1A4, Canada; 3 Nanthana-Kriangkrai Chotiwattanaphan (NKC) Institute of Gastroenterology and Hepatology, Faculty of Medicine, Prince of Songkla University, Hat Yai, Songkhla 90110, Thailand; nisasan@yahoo.com (N.N.); rochet7488@hotmail.com (S.K.); 4 Department of Internal Medicine and Oncology, Semmelweis University, 1085 Budapest, Hungary

**Keywords:** vonoprazan, potassium-competitive acid blocker, proton pump inhibitors, peptic ulcer, bleeding

## Abstract

**Background**: Proton pump inhibitor (PPI) therapy is well-established for its effectiveness in reducing re-bleeding in high-risk peptic ulcer patients following endoscopic hemostasis. Vonoprazan (VPZ) has demonstrated the capacity to achieve gastric pH levels exceeding 4, comparable to PPIs. This study aims to evaluate the comparative efficacy of intravenous PPI infusion versus VPZ in preventing re-bleeding after endoscopic hemostasis in patients with high-risk peptic ulcers. **Methods**: A randomized, double-blind, controlled, and double-dummy design was employed. Patients with peptic ulcer bleeding (Forrest class IA/IB or IIA/IIB) who underwent endoscopic hemostasis were randomly assigned to either the PPI group or the VPZ group. Re-bleeding rates at 3, 7, and 30 days, the number of blood transfusions required, length of hospitalization, and ulcer healing rate at 56 days were assessed. **Results:** A total of 44 eligible patients were enrolled, including 20 patients (PPI group, *n* = 11; VPZ group, *n* = 9) with high-risk peptic ulcers. The mean age was 66 years, with 70% being male. Re-bleeding within 72 h occurred in 9.1% of the PPI group versus 0% in the VPZ group (*p* = 1.000). There was no significant difference in re-bleeding rates within 7 days and 30 days (18.2% vs. 11.1%, *p* = 1.000). Additionally, the ulcer healing rate did not significantly differ between the groups (87.5% vs. 77.8%). **Conclusions**: This pilot study demonstrates comparable efficacy between oral vonoprazan and continuous PPI infusion in preventing recurrent bleeding events among high-risk peptic ulcer patients following successful endoscopic hemostasis.

## 1. Introduction

Peptic ulcer bleeding stands as a frequent cause of hospital admissions, placing a substantial strain on healthcare systems globally. Mortality rates are subject to a multitude of influences, encompassing recent hemorrhage indicators, the proficiency of the performing endoscopist, recurrence of bleeding episodes, the administration of intravenous proton pump inhibitors (PPIs), and the presence of significant comorbidities [1,2,3,4]. Despite the widespread adoption of endoscopic therapy and acid suppression as primary treatment strategies, the incidence of re-bleeding within 72 h remains notably high, with rates ranging from 4% to 7% following endoscopic intervention [5,6,7,8].

Multiple studies have demonstrated the efficacy of intravenous PPIs in reducing recurrent bleeding in high-risk peptic ulcer patients who have undergone endoscopic hemostasis [6]. Therefore, the management guidelines for non-variceal upper gastrointestinal bleeding recommend that such patients receive a continuous intravenous PPI at a high dose for 72 h, or a high-dose PPI twice daily, followed by 14 days of a standard-dose PPI for a minimum of 8 to 12 weeks to decrease the risk of recurrent bleeding [2,9]. Several studies have further shown that maintaining an intragastric pH above 6 is associated with protecting clot integrity, enhancing platelet aggregation, and thereby preventing re-bleeding [7,10,11].

Vonoprazan represents a novel potassium-competitive acid blocker (P-CAB), belonging to a class of competitive potassium inhibitors that reversibly inhibit the gastric acid pump through K+-competitive mechanisms. Unlike proton pump inhibitors (PPIs), which rely on acid activation, P-CABs like vonoprazan inhibit the enzyme through reversible K+-competitive ionic binding. Vonoprazan exhibits rapid and prolonged acid suppression compared to PPIs, with PPIs typically requiring approximately 3–5 days to achieve maximal gastric acid suppression [12,13]. Several studies have demonstrated that vonoprazan is non-inferior to oral PPIs in acid suppression, as evidenced by pH over four holding time ratios [13,14]. Kagawa et al. reported that vonoprazan significantly reduces post-endoscopic submucosal dissection (ESD) bleeding and promotes better ulcer healing rates compared to PPIs [15]. Furthermore, the efficacy of vonoprazan extends to the treatment of acid-related diseases such as erosive esophagitis, healing post-ESD ulcers, and Helicobacter pylori eradication [15,16,17,18]. However, the efficacy of vonoprazan in the treatment of post-endoscopic hemostasis has not yet been demonstrated.

In the present study, our aim was to conduct a randomized, double-blind, controlled trial to assess the effectiveness of vonoprazan compared to intravenous PPI infusion in preventing re-bleeding in patients with high-risk peptic ulcer bleeding following endoscopic hemostasis. We compared the rates of recurrent bleeding, ulcer healing, and adverse events between these two treatment groups.

## 2. Materials and Methods

### 2.1. Study Design and Patients

We conducted a prospective, double-blinded, double-dummy, randomized controlled study involving adult patients aged 18 to 85 years with high-risk stigmata of peptic ulcer bleeding, as defined by Forrest IA/IB and IIA/IIB classification, between February 2021 and February 2023. The study was conducted at the NKC Institute and Songklanagarind Hospital, Faculty of Medicine, Prince of Songkla University, Hat Yai, Songkhla, Thailand.

All patients underwent endoscopic hemostasis procedures, including thermal coagulation and hemoclipping, to achieve successful hemostasis. Exclusion criteria encompassed severe comorbidity (American Association class 3–4) at presentation, advanced-stage malignancy, decompensated cirrhosis (Child Turcotte Pugh score class C), failure to achieve successful endoscopic hemostasis, coagulopathy, inability to commence oral feeding within 6 h post-endoscopic intervention, and a history of allergy to vonoprazan or PPI. Ethical approval was obtained from the ethics committee, and the study was registered at www.clinicaltrials.in.th, accessed on 12 May 2024 (TCTR20210227002).

Following successful endoscopic hemostasis, patients were randomly allocated to one of two groups: (I) the PPI group, where patients received a pantoprazole infusion at 8 mg per hour for 72 h and oral placebo every 12 h; or (II) the VPZ group, where patients received oral vonoprazan at a dose of 20 mg every 12 h and a placebo infusion for 72 h.

**Figure 1:**
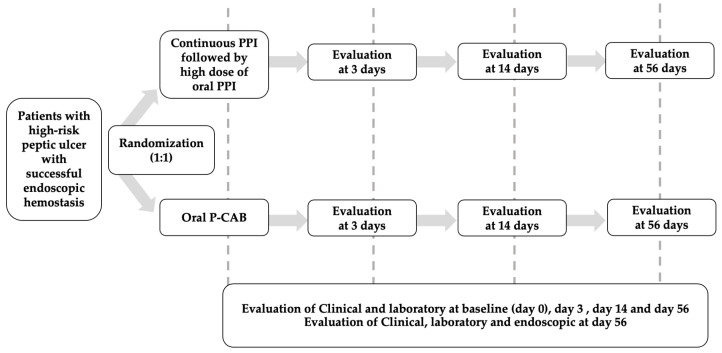
Study protocol.

After the initial 72 h of treatment, patients in the PPI group received oral omeprazole at a dose of 20 mg every 12 h, along with an oral placebo every 12 h, from day 3 to day 14. Subsequently, they were transitioned to oral omeprazole at a dose of 20 mg once daily, accompanied by an oral placebo once daily, from day 15 to day 56. In the VPZ group, patients were administered oral vonoprazan at a dose of 20 mg every 12 h, in addition to an oral placebo every 12 h, from day 3 to day 14. Then, they received vonoprazan at a dose of 20 mg once daily, along with an oral placebo, from day 15 to day 56. The study protocol is depicted in Figure 1.

Randomization was conducted using a computer-generated sequence by blocks of four, and the study groups were blinded using sealed consecutively numbered envelopes within a sealed box. The PPI group received intravenous pantoprazole (Takeda Pharmaceutical Co., Tokyo, Japan) and oral omeprazole (Thai Government Pharmaceutical Organization), while the VPZ group received vonoprazan (Takeda Pharmaceutical Co., Tokyo, Japan). The placebo medications were designed to closely resemble the shape and color of oral omeprazole and vonoprazan for easy identification. Additionally, the intravenous placebo was formulated using normal saline solution and packaged in sealed containers.

### 2.2. Endoscopic Hemostasis Treatment

Endoscopic hemostasis procedures were conducted according to the expertise and experience of three endoscopists, each with a minimum of 5 years of experience in therapeutic interventions for upper gastrointestinal bleeding. In our study, endoscopy was performed using a GIF-1TH190 endoscope manufactured by Olympus Medical System, CORP., Tokyo, Japan.

The standard endoscopic treatment for bleeding peptic ulcers comprised the use of contact methods, such as a bipolar electrohemostasis catheter (Gold probe^TM^, Boston Scientific Corporation, Marlborough, MA, USA), as well as non-contact thermocoagulation (argon plasma coagulation) or hemoclipping, with or without prior injection of diluted epinephrine. Ulcer size was evaluated using forceps biopsy (Boston Scientific Corporation, Marlborough, MA, USA) prior to endoscopic intervention (the maximum diameter of the biopsy forceps was 6 mm).

Successful endoscopic hemostasis was defined as the cessation of active bleeding or the disappearance of high-risk stigmata of bleeding during observation for at least 3 min. Patients with uncontrolled bleeding despite standard endoscopic methods were referred for angiographic embolization or surgery. In patients with a high risk of cardiovascular or thromboembolic events, the timing of resuming antiplatelet or anticoagulation therapy was determined by the treating cardiologist.

### 2.3. Study Outcomes

The primary endpoint of our study was the re-bleeding rate within 72 h following endoscopic hemostasis. Secondary endpoints included the rate of re-bleeding within 30 days and the complete healing of ulcers at 56 days post-endoscopic hemostasis.

Re-bleeding was defined as the presence of recurrent hematemesis, passage of melena, and/or fresh blood staining in the nasogastric tube after documented successful endoscopic hemostasis, in addition to any of the following criteria: (i) hypotension (systolic blood pressure lower than 90 mm Hg) or tachycardia (pulse rate more than 120/min) without other explained causes; (ii) a decrease in hemoglobin of more than 2 g/dL during any 24 h period; or (iii) receiving at least 2 units of blood transfusion within 72 h. Patients experiencing recurrent bleeding underwent endoscopic confirmation, which revealed stigmata of recent hemorrhage. Complete ulcer healing was defined as the absence of an ulcer at follow-up endoscopy at 56 days. Incomplete ulcer healing was defined as the persistence of the ulcer or partial healing without complete resolution.

### 2.4. Statistical Analysis

Baseline demographic characteristics and categorical variables were presented as frequencies with percentages and compared using either the χ^2^ square or Fisher’s exact tests. Continuous variables were compared between the two groups using Wilcoxon’s test for non-normally distributed data and Student’s t-test for normally distributed data. Categorical data were compared using the chi-square test or Fisher’s exact test.

Follow-up duration was calculated based on the date of endoscopic hemostasis. Patients were categorized into the VPZ and PPI groups. The efficacy outcomes were analyzed using a per-protocol analysis. Recurrent bleeding probability was demonstrated using Kaplan–Meier survival curves and the Peto and Peto test was utilized for comparison of statistical significance between the groups. A *p*-value < 0.05 was considered statistically significant. All statistical analyses were performed using the R program version 4.1.0 (R Foundation for Statistical Computing, Vienna, Austria).

## 3. Results

**Figure 2:**
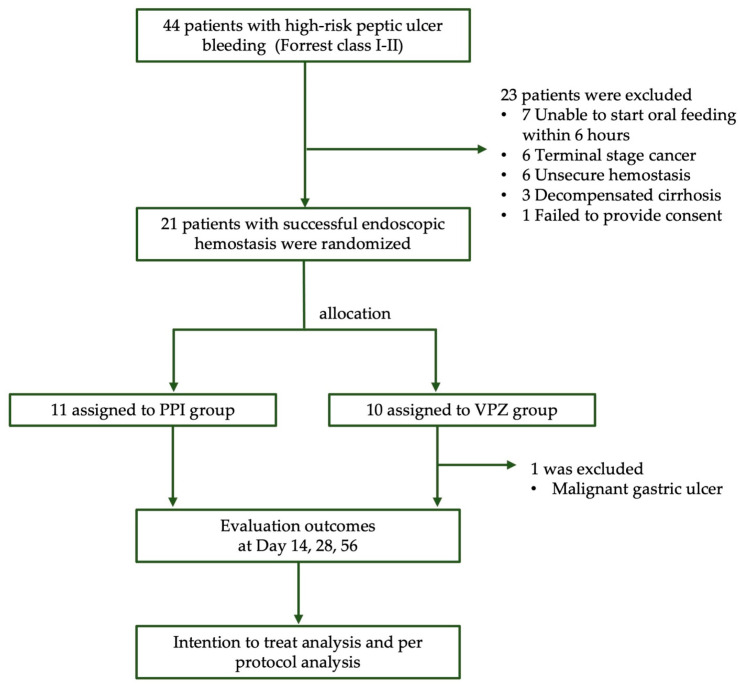
Study flow chart and randomization.

A total of 44 patients were consecutively enrolled, with 23 patients subsequently excluded (Figure 2). Of the remaining participants, 20 patients (11 in the PPI group and nine in the VPZ group) were included in the efficacy analysis. The mean age of the cohort was 66 years, with 70% being male. One patient in the VPZ group was excluded from the analysis due to a pathology-proven diagnosis of gastric adenocarcinoma.

There were no significant differences observed in age, sex, ASA classification, comorbidities, or the use of antiplatelet or anticoagulant medications between the VPZ and PPI groups. Additionally, baseline clinical severity scores, including the Glasgow–Blatchford score (GBS), Rockall score, and AIMS-65 score, were not significantly different between the two groups. Table 1 summarizes the baseline characteristics of the patients in the study.

The majority of ulcers (75%) were duodenal ulcers, with 75% of them classified as Forrest 2a. Although the ulcer size was numerically larger in the PPI group (10 mm) compared to the VPZ group (6 mm), this difference did not reach statistical significance. Coagulation techniques (thermal or argon plasma) were utilized for endoscopic hemostasis in 65% of patients, while hemoclipping was employed in 10% of patients. The rapid urease test for Helicobacter pylori was positive in 40% of patients. Further details regarding endoscopic hemostasis and Helicobacter pylori status are provided in Table 2.

**Figure 3:**
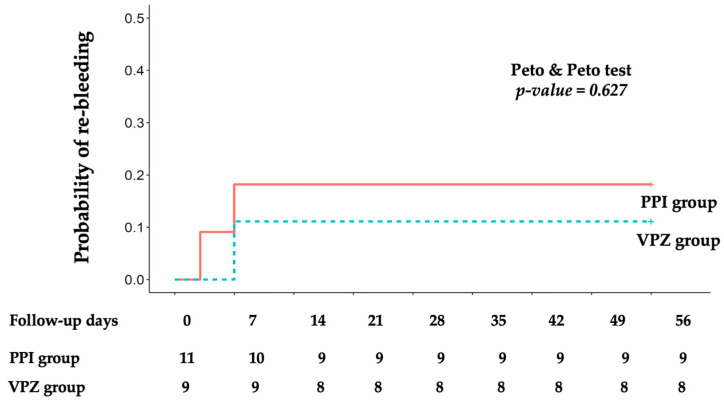
Kaplan–Meier curve for probability of clinical peptic ulcer re-bleeding.

There were no significant differences observed in the primary outcomes, with the rates of recurrent bleeding within 7 days (18.2% vs. 11.1%, *p* = 1.000) and 30 days (18.2% vs. 11.1%, *p* = 1.000) showing no statistically significant difference. Only one patient in the PPI group experienced re-bleeding within 72 h after endoscopic hemostasis. The probability of clinical re-bleeding did not show a significant difference between the two groups (Peto and Peto test *p*-value = 0.627) (Figure 3).

The secondary outcomes, particularly the rates of completed ulcer healing, appeared numerically higher in the VPZ group compared to the PPI group, without reaching statistical significance (87.5% vs. 77.8%, *p* = 1.000). Six patients showed ulcer improvement (smaller size) but not complete resolution. The length of hospital stay was similar between both groups (5 days each, *p* = 0.451). There were no reported incidences of serious adverse events (SAEs) within the study cohort. Clinical outcomes regarding re-bleeding and ulcer healing rates are detailed in Table 3.

## 4. Discussion

Recurrent bleeding from peptic ulcers poses significant morbidity and mortality risks, particularly among elderly patients with multiple comorbidities [19]. Re-bleeding typically occurs within 3–7 days following endoscopic intervention. The standard protocol for facilitating negotiated healing and achieving hemostasis involves a combination of endoscopic intervention and high-dose proton pump inhibitors (PPIs). The European Society of Gastrointestinal Endoscopy (ESGE) Guidelines recommend the administration of high-dose PPIs, either via continuous infusion or intravenous bolus, for a duration of 72 h following endoscopic hemostasis, serving as alternative regimens [9].

This study is a randomized, double-blind, placebo-controlled trial aimed at comparing the efficacy of an intravenous continuous PPI versus an oral potassium-competitive acid blocker (P-CAB) in preventing recurrent bleeding from peptic ulcers in high-risk patients who have undergone endoscopic treatment. Our findings indicate that there was no significant difference in recurrent bleeding rates between the high-dose PPI and oral vonoprazan (40 mg/day) at 3, 7, 30, and 56 days following successful endoscopic hemostasis.

Our study assessed several crucial pre-endoscopic risk stratification tools, including GBS, AIMS-65, and Rockall scores, to predict recurrent bleeding, mortality, and the necessity for endoscopic interventions [20]. Of note, our cohort comprised patients at a very high risk for re-bleeding, with a mean GBS score of 13 and a Rockall score of 6.5. Previous reports have indicated that a GBS score greater than 7.5 (with a sensitivity of 92.9% and specificity of 52.9%) or a Rockall score greater than 4.5 (with a sensitivity of 92.9% and specificity of 57.5%) are associated with high mortality [21].

In our study, the rate of re-bleeding within 72 h after endoscopic hemostasis was 10%, which is higher than the rates reported in earlier studies (ranging from 4–5.9%) [5,7,8]. This disparity can be attributed to several factors present in our cohort, including advanced age, co-existing comorbidities, and a higher proportion of patients using antiplatelet or anticoagulant medications in the context of a referral center setting. Sung et al. reported a re-bleeding rate of 5.1% in peptic ulcer patients; however, their study excluded patients unable to discontinue dual antiplatelet therapy [7]. In contrast, in our study, one patient who experienced early re-bleeding within 72 h had to resume dual antiplatelet therapy for acute coronary syndrome. Additionally, three patients who experienced re-bleeding within 7 days resumed dual antiplatelet or anticoagulant therapy due to cardiopulmonary issues, while one patient required hemodialysis.

The strength of our present study lies in its randomized controlled, double-dummy blinded design. We report the favorable efficacy of vonoprazan in preventing re-bleeding post-endoscopic hemostasis in patients with high-risk peptic ulcers. For clinical practice implications, given its oral route of administration, vonoprazan may serve as an alternative to intravenous PPIs as an acid inhibitor following successful endoscopic hemostasis treatment. Furthermore, all patients in our study received follow-up esophagogastroduodenoscopy (EGD) for re-evaluation of ulcer healing 56 days after endoscopic intervention. Nevertheless, there were limitations to our study. First, the number of enrolled patients was relatively small due to difficulties in enrollment during the COVID-19 pandemic, and many patients received pre-endoscopic infusion PPIs. This may have obscured the stigmata of recent hemorrhage, resulting in fewer high-risk peptic ulcer identifications. Therefore, the results of this pilot study require further validation with a larger patient cohort to draw solid conclusions. Second, patients who could not start oral feeding within 6 h after successful endoscopic hemostasis were excluded, limiting the application of vonoprazan to those able to take oral medication. Lastly, we did not measure intragastric pH during the study.

## 5. Conclusions

This pilot study demonstrates comparable efficacy between oral vonoprazan and continuous PPI infusion in preventing re-bleeding in patients with high-risk peptic ulcers following successful endoscopic hemostasis. Further larger studies are needed to confirm the efficacy of vonoprazan in preventing post-endoscopic re-bleeding.

## Figures and Tables

**Table 1:** Baseline characteristics of the study patients.

Characteristics	All Cohort (*n* = 20)	PPI Group (*n* = 11)	VPZ Group (*n* = 9)	*p*-Value
Gender: male, *n* (%)	14 (70)	8 (72.7)	6 (66.7)	1.000
Mean age: years (SD)	66.4 (14.9)	64.9 (17.1)	68.2 (12.5)	0.634
Mean BMI, kg/m^2^ (SD)	23.3 (4.3)	22.2 (4)	24.5 (4.6)	0.254
ASA classification, *n* (%)				0.540
0	3 (15)	2 (18.2)	1 (11.1)	
I	2 (10)	0	2 (22.2)	
II	13 (65)	8 (72.7)	5 (55.6)	
III	2 (10)	1 (9.1)	1 (11.1)	
Cardiovascular disease, *n* (%)	11 (55)	5 (45.5)	6 (66.7)	0.406
Chronic kidney disease, *n* (%)	2 (10)	2 (18.2)	1 (11.1)	0.227
Active smoking, *n* (%)	4 (20)	3 (27.3)	1 (11.1)	0.678
NSAIDs user, *n* (%)	11 (55)	6 (54.5)	5 (55.6)	1.000
Antiplatelet user, *n* (%)	10 (50)	5 (45.5)	5 (55.6)	1.000
Anticoagulant, *n* (%)	3 (15)	1 (9.1)	2 (22.2)	0.566
PPI user, *n* (%)	2 (10)	1 (9.1)	1 (11.1)	1.000
BUN, mg/dL (IQR)	34 (27.4–72)	30.1 (26.5–76.8)	37.7 (27.4–72)	0.649
Hemoglobin, mg/dL (SD)	7.2 (1.4)	7.7 (1.4)	6.7 (1.4)	0.150
Albumin, mg/dL (SD)	3.3 (0.6)	3.4 (0.7)	3.1 (0.5)	0.430
Anemic symptoms, *n* (%)	15 (75)	7 (63.6)	8 (88.9)	0.319
Glasgow blatchford score (SD)	13.3 (2.8)	13.1 (2.8)	13.6 (3)	0.727
AIMS 65 score (SD)	1.4 (0.9)	1.5 (0.9)	1.4 (1)	0.982
Rockall score (SD)	6.4 (1.7)	6.4 (1.7)	6.6 (1.7)	0.809
Time to endoscopy, hours (SD)	18.8 (10)	19.5 (10.3)	18 (10.1)	0.748

Abbreviations: NSAIDs: nonsteroidal anti-inflammatory drugs, PPI: proton pump inhibitor, VPZ: vonoprazan; ASA: The American Society of Anesthesiologists (ASA) physical status classification; SD: standard deviation.

**Table 2:** Characteristics of peptic ulcer and endoscopic hemostasis.

Variables	All Cohort (*n* = 20)	PPI Group (*n* = 11)	VPZ Group (*n* = 9)	*p*-Value
Ulcer size, mm (IQR)	7 (5–15)	10 (5–19)	6 (6–15)	0.7
Ulcer locations, *n* (%)				
Antrum	5 (25)	2 (18.2)	3 (33.3)	0.617
Duodenal bulb	15 (75)	9 (81.8)	6 (66.7)	0.617
Forrest classification, *n* (%)				
- IA	2 (10)	1 (9.1)	1 (11.1)	1
- IB	1 (5)	1 (9.1)	0	1
- IIA	15 (75)	9 (81.8)	6 (66.7)	0.617
- IIB	2 (10)	0	2 (22.2)	0.189
Number of PRC transfusion, unit (SD)	2.4 (1.1)	2.5 (1.1)	2.2 (1.2)	0.662
Endoscopic hemostasis, *n* (%)				
- Epinephrine injection	17 (85)	9 (81.8)	8 (88.9)	1.000
- Thermal coagulation	13 (65)	9 (81.8)	4 (44.4)	0.16
- Argon plasma coagulation	4 (20)	1 (9.1)	3 (33.3)	0.285
- Hemoclip	2 (10)	2 (18.2)	2 (22.2)	0.811
Helicobacter pylori infection, *n* (%)	8 (40)	6 (54.5)	2 (22.2)	0.197
Procedure time, min (IQR)	40 (28.8–45)	40 (27.5–42.5)	40 (30–45)	0.564

**Table 3:** Clinical outcomes and re-bleeding rates.

Outcomes	All Cohort (*n* = 20)	PPI Group (*n* = 11)	VPZ (*n* = 9)	*p*-Value
Primary outcome				
Re-bleeding within 72 h, *n* (%)	1 (5)	1 (9.09)	0	1.000
Secondary outcome				
Re-bleeding within 7 days, *n* (%)	3 (15)	2 (18.2)	1 (11.1)	1.000
Re-bleeding within 30 days, *n* (%)	3 (15)	2 (18.2)	1 (11.1)	1.000
Length of hospital stay, days (IQR)	5 (4–6.2)	5 (4.5–6.5)	5 (4–5)	0.451
Completed ulcer healing at day 56, *n* (%)	14 (82.4)	7 (77.8)	7 (87.5)	1.000

## Data Availability

The data underlying this article come from those included in the relevant published articles. The latter will be shared upon reasonable request to the corresponding author.
